# The Impact of Digital Technology on the Physical Health of Older Workers: Scoping Review

**DOI:** 10.2196/78406

**Published:** 2025-11-18

**Authors:** Jeroen JA Spijker, Hande Barlın, Melina Dritsaki, Yang Gu, Aija Klavina, Nilufer Korkmaz Yaylagul, Gunilla Kulla, Murat Anil Mercan, Eda Orhun, Anna Sevcikova, Brigid Unim, Gunay Yildizer, Cristina Maria Tofan

**Affiliations:** 1 Institute for Advanced Family Studies Universitat Internacional de Catalunya Barcelona Spain; 2 Centre for Demographic Studies (CED-CERCA) Bellaterra Spain; 3 Department of Economics Gebze Technical University Gebze Turkey; 4 Department of Economics University of Western Macedonia Kastoria Greece; 5 Department of Work, Employment, Management & Organization University of Leicester Leicester United Kingdom; 6 Laboratory of Sports and Nutrition Research Riga Stradiņš University Riga Latvia; 7 Department of Health Promotion and Rehabilitation Lithuanian Sports University Kaunas Lithuania; 8 Klaipėdos Valstybinė Kolegija / Higher Education Institution Klaipeda Lithuania; 9 Department of Gerontology Faculty of Health Sciences Akdeniz University Antalya Turkey; 10 Department of Health and Caring Sciences Western Norway University of Applied Sciences Forde Norway; 11 Department of Economics Yıldız Technical University Istanbul Turkey; 12 School of Business Al Akhawayn University Ifrane Morocco; 13 Faculty of Social Studies Masaryk University Brno Czech Republic; 14 Department of Cardiovascular, Endocrine-Metabolic-Diseases and Ageing Italian National Institute of Health Rome Italy; 15 Faculty of Sport Sciences Eskisehir Technical University Eskişehir Turkey; 16 Psychology and Educational Sciences Department “Gheorghe Zane” Institute for Economic and Social Research Romanian Academy Iasi Romania; 17 Department of Sociology, Social Work and Human Resources Faculty of Philosophy and Social-Political Sciences Alexandru Ioan Cuza University Iasi Romania

**Keywords:** digital tools, digital technology, physical health, mobility, vision loss, musculoskeletal disorders, older workers, older population, aging, scoping review

## Abstract

**Background:**

Digital technologies are increasingly present in workplaces; however, their impact on the physical health of older workers remains unclear.

**Objective:**

This scoping review aims to examine and summarize the scientific evidence on how digital technology affects the physical health of older workers.

**Methods:**

Following PRISMA-ScR (Preferred Reporting Items for Systematic Reviews and Meta-Analysis extension for Scoping Reviews) guidelines, we conducted a scoping review of English-language peer-reviewed studies extracted from MEDLINE, Cochrane, ProQuest, Web of Science, Scopus, APA PsycInfo, and ERIH PLUS. The review followed the population, concept, and context (PCC) framework, including studies on workers aged 50 years or older, any form of digital technology (eg, teleworking and the use of digital tools at work), and its impact on physical health (eg, vision loss and musculoskeletal disorders). Studies that focused only on mental health were excluded. A 13-member research team screened studies in 3 stages, namely title and abstract screening, full-text review, and data extraction. Each study was independently reviewed by at least 2 researchers, and disagreements were resolved through discussion. Data extraction and synthesis were conducted using the web-based systematic review platform Covidence (Veritas Health Innovation Ltd).

**Results:**

In total, 18 studies were selected, published between 2012 and 2024, with most conducted in Europe (n=8) and Asia (n=6), followed by North America (n=2), Oceania (n=1), and Africa (n=1). We identified 6 key physical health areas impacted by digital technology in older workers, including eye health, musculoskeletal health, metabolic and cardiovascular health, workplace sound levels, and user experiences of new technologies. Findings showed mixed effects, with notable negative impacts on eye strain, musculoskeletal disorders, and hearing health issues, but positive effects on weight management, cardiovascular health, physical activity, and perceived physical well-being.

**Conclusions:**

Digital technology presents both risks and benefits for the physical health of older workers. While prolonged screen use and digital work environments contribute to eye strain, musculoskeletal issues, and hearing concerns, other technologies support better weight management, cardiovascular health, and increased physical activity. These findings also underscore the need for workplace intervention to reduce health risks.

**International Registered Report Identifier (IRRID):**

RR2-10.2196/59900

## Introduction

Physical health can be defined as a “dynamic state, the process of preserving and developing its biological, physiological, and mental functions, optimal work capacity, and social activity with the maximum life expectancy” [1]. During the last decade, research literature presents growing concern not only in the context of individual aging but also in workplace aging, as working life and retirement age continue to extend [2]. For older workers, maintaining physical health involves not only performing job-related tasks effectively but also preventing and managing age-related conditions to support overall well-being. Significant physiological changes associated with aging affect various capabilities, including sensory function, muscle function, cardiovascular and respiratory function, neurological function, and immune response [3]. However, despite the inevitable age-related physiological decline, a significant proportion of older workers demonstrate adaptation abilities that enable them to maintain work performance [4].

From 2004 to 2019, the share of workers aged 55 years and older in the European Union (EU) workforce increased from 12% to 20% [5]. However, 21.6% of these employees reported more than 2 work-related physical health problems [6]. As the global workforce ages and digital technologies increasingly penetrate the occupational landscape, new job roles and work conditions have emerged. The labor market and traditional work processes have undergone significant transformations through practices, such as remote work, blended work, teleworking, and the use of digital applications, including mobile apps. These changes have introduced new physical and psychological demands [7], bringing both advantages and disadvantages. For instance, digital tools offer greater flexibility, enabling employees to work from home and reduce reliance on physically demanding tasks, thereby lowering the risk of musculoskeletal injuries. However, these benefits can only be realized if workstations are ergonomically designed and regular breaks are taken [8].

Physical health has always been a concern for older adults, but with extended working lives and delayed retirement, it is increasingly critical for older workers. A large study examining work-related health risks among older and younger workers found that perceptions of risk differ by age group, often deviating from actual exposure (eg, injury) [6]. Additionally, workplace digitalization presents new physical health challenges for older workers, such as prolonged screen exposure causing eye strain and headaches, extended periods of sitting without physical activity increasing the risk of cardiovascular diseases, and the stress of constant connectivity contributing to spinal and postural issues [8-10].

Moreover, difficulty adapting to technology also affects older workers’ health and productivity. Therefore, continuous training, regular biometric and physical health monitoring [11,12], maintaining fitness, managing chronic diseases, and workplace adaptations are key strategies to enhance the safety, health, and productivity of older workers. Likewise, digital health training programs can assist older employees in maintaining health through retirement transition, positively influencing physical well-being [13].

Despite an aging workforce and the digital transformation of work environments, comprehensive research on how digitalization affects the physical health of older workers remains limited. Older workers are often assumed to be more affected than their younger peers [14]. While some recent studies have explored the psychological consequences of workplace digitalization—technostress [15,16], burnout, and psychological strain [11,17,18]—as well as the role of digital technologies in health and disease management [6,12,13,19-24], the physical health consequences for older employees remain largely unexplored. This scoping review aims to bridge that gap by analyzing existing research on this crucial health issue in older workers.

## Methods

### Guidance Frameworks

To guarantee a systematic and coherent process, this scoping review followed the recommendations of Levac et al [25], which were built on the Arksey and O’Malley [26] framework. The process included the following stages: (1) identifying the research question, (2) identifying relevant studies, (3) selecting studies, (4) charting and collating the data, and (5) summarizing and reporting the results. The findings are reported according to the PRISMA-ScR (Preferred Reporting Items for Systematic Reviews and Meta-Analysis extension for Scoping Reviews) guidelines [27,28].

### Protocol and Registration

The protocol was published [27] and registered under the International Registered Report Identifier (IRRID): PRR1–10.2196/59900 [28] before conducting the full electronic literature search. A search for existing scoping reviews found no studies on the impact of digitalization on physical health, though several focused on its effects on mental health [27]. No changes were made between the published protocol and this scoping review.

### Stage 1: Identifying the Research Question

We posed the main research question, “How do digital technologies in the workplace affect the physical health of older workers?” Given the wide range of ways in which digital technology can affect an older worker’s physical health (from vision loss to musculoskeletal disorders) and the diversity of workplace digitalization (from working online from home to the use of robotics in a car assembly line), we formulated the following subquestions:

1. What are the most common physical health issues that older workers face as a result of workplace digitalization?

2. Which industries most strongly affect older workers’ physical health as a result of workplace digitalization?

To refine our focus, we applied the population, concepts, and context (PCC) framework [25] (Multimedia Appendix 1 [29,30]). In summary, our review examines studies on older workers aged 50 years and older (Population). Digital technologies (Concept) refer to electronic devices or tools used for data manipulation, storage, and transmission, specifically in work-related activities. The Context focuses on how these technologies impact physical health, excluding nonwork or nondigital applications.

### Stage 2: Identifying Relevant Studies

Following the Joanna Briggs Institute Manual for Evidence Synthesis [31] search strategy, we searched for peer-reviewed studies written in English in the following databases: MEDLINE, Cochrane, ProQuest, Web of Science, Scopus, APA PsycInfo, and ERIH PLUS. We used the web-based systematic review platform Covidence (Veritas Health Innovation Ltd) to import the references and remove duplicates. The search terms used are listed in Textbox 1.

**Search terms.**

(physical adj (health* OR condition* OR issue* OR impairment* OR fitness OR wellbeing OR (well adj being) OR integrit* OR state* OR stress) OR disease* OR vision OR mobility OR obes* OR overweight OR “Body Mass Index”) AND (digital OR app* OR web OR internet OR tech* OR (social adj media) OR chat OR online* OR cyber OR virtual OR computerized OR computerised OR electronic OR ICT) AND ((old* or elder* or ageing or ageing or senior*) adj1 (work* OR employee* OR profession* OR labor OR labour OR colleague* OR staff* OR cowork* OR personnel)).

### Stage 3: Study Selection and Eligibility Criteria

A total of 7290 references were imported into Covidence for screening. After removing 461 duplicates, 6638 studies proceeded to title and abstract screening, followed by a full-text review. Through this process, 17 relevant studies addressing the main and secondary research questions were selected [32-48]. Additionally, one more study was added post hoc, as Covidence did not identify it due to the use of “middle-aged” instead of (synonyms for) “older workers” in the title and abstract, despite the sample age range, including individuals aged 35-65 years [9]. Given the lack of a standard definition of “older workers,” we included studies with participants aged 50 years and older, even if younger workers were also part of the sample and analyzed (eg, age group >45 years). Similarly, we included studies across diverse occupational settings, ranging from health care professionals and office workers to farmers and self-employed individuals, as well as studies examining a wide variety of digital technologies. For the purpose of this review, we considered both studies that explicitly examined digital tools (eg, personal computers, smartphones, and specialized apps) and those where technology use was implicit in the work arrangement (eg, telework). This broad approach ensured that potentially relevant evidence was not excluded, though it introduced heterogeneity in populations, contexts, and technologies. A detailed overview of the study characteristics, including design, sample, occupational setting, and type of digital technology examined, is provided in Multimedia Appendix 2 for ease of reference.

All coauthors participated in the selection process, with each study reviewed by at least 2 researchers. Conflicts identified by Covidence regarding study inclusion or exclusion were resolved through discussion or by consulting a third reviewer.

Eligibility was determined based on the PCC framework and additional criteria shown in Textbox 2, while the selection process is synthesized in a PRISMA (Preferred Reporting Items for Systematic Reviews and Meta-Analysis) flowchart. Notably, Covidence allows only one exclusion reason per record during the full-text screening phase. However, many excluded studies met multiple exclusion criteria (eg, lacking both relevant physical health outcomes and age-specific data), which is why only the categories of exclusion—not the number of studies per reason—are reported in the flowchart.

No restrictions were placed on publication year or study location, as the absence of a prior scoping review on this specific topic in a peer-reviewed journal justified including all years, while excluding countries would have required further justification.

**Eligibility criteria for the scoping review.**

**Inclusion criteria**

- Population: older workers (participants more than 50 years of age were included in the study)
- Concept: digital technologies or tools related to work
- Context: physical health outcomes
- Setting: nonclinical and in the work sphere
- Study type: original studies with any design or data type (quantitative, qualitative, and mixed)
- Publication status: published in a peer-reviewed journal
- Publication language: English
- Full text available

**Exclusion criteria**

- Population: younger workers (participants more than 50 years of age were not included in the study)
- Concept: digital technologies or tools not related to work (eg, for health management)
- Context: non-physical health outcomes (eg, mental health)
- Setting: clinical and not in the work sphere
- Study type: other study types (eg, protocols, narrative reviews, or systematic reviews)
- Publication status: published without peer review, dissertations, books, conference papers, letters, and editorials.
- Publication language: written in a language other than English
- Full text not available

### Stages 4 and 5: Charting the Data and Collating, Summarizing, and Reporting Results

From each selected publication in the final Covidence phase, the main outcomes of interest were extracted, including bibliographic information, study designs and aims, population characteristics, the type of digital technology or tools analyzed (concept), the type of physical health addressed (context), and the main findings (Multimedia Appendix 2). These findings were analyzed, evaluated, and discussed following the PRISMA-ScR [49] guidelines.

We assessed study quality using the Mixed Methods Appraisal Tool (MMAT) [50], which evaluates five study design categories: (1) qualitative studies, (2) quantitative randomized controlled trials, (3) quantitative nonrandomized studies, (4) quantitative descriptive studies, and (5) mixed methods studies. The MMAT includes a 2-part checklist: an initial screening with 2 questions to confirm the study’s empirical nature (clarity of research questions and feasibility of addressing them), followed by 5 design-specific criteria guiding the assessment process.

Three reviewers independently appraised each study to minimize bias and ensure reliability. Discrepancies were resolved through discussion. Each study was assessed using 2 initial screening questions (“Yes”=1, “No”=0) and 5 design-specific criteria (“Yes”=1, “No”=0, “Don’t know”=0), evaluating methodology, data collection, relevance of measurements, and coherence between data sources and analysis methods. We subsequently calculated the percentage of “Yes” responses for each study to assess methodological quality, and no studies were excluded based on this assessment. The quality score was calculated by dividing the number of “Yes” responses by the total applicable criteria (2 for screening, 5 for study design) and multiplying by 100. These scores (0%-100%) represent the proportion of MMAT criteria met. We then averaged quality scores across the screening items and each study design category to provide an overview. This quantitative measure contextualizes the reliability and validity of the findings within our review.

## Results

### Qualitative Assessment

**Figure 1:**
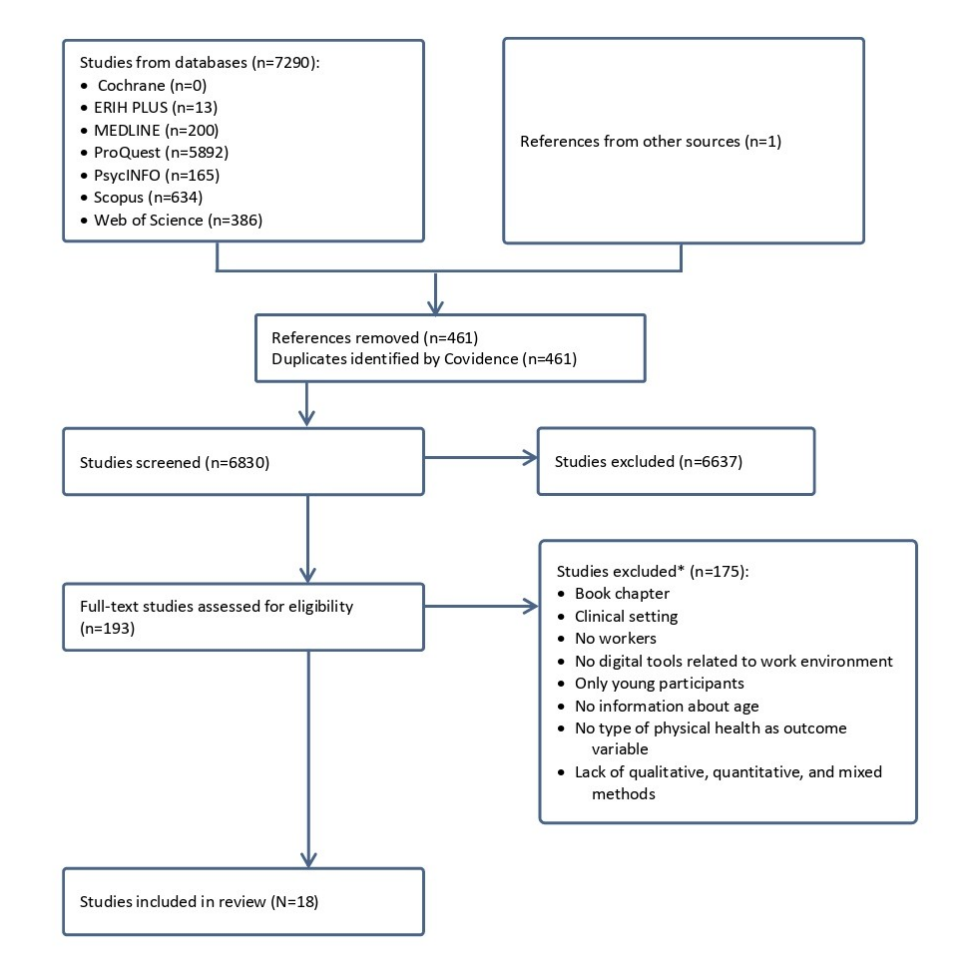
PRISMA flowchart of studies through the scoping review process. *No values provided, as Covidence allows only one reason per entry while there were often several reasons to exclude studies.

All 18 selected studies (Figure 1) stated clear research questions and collected appropriate data to address them (Multimedia Appendix 3). Although not all studies explicitly described their study design, we identified 9 quantitative nonrandomized studies based on the MMAT user guide [50], with an average quality score of 76% (SD 19%) on the MMAT criteria. These included 1 prospective study [32], 3 cohort studies [33-35], 3 cross-sectional studies [9,36,37], 1 quasi-experimental study [38], and 1 retrospective observational study [39]. Furthermore, we identified 4 quantitative descriptive studies (mean score 65%, SD 30%), including 2 prevalence studies [40,41] and 2 survey-based studies [42,43], 2 quantitative randomized controlled trials (mean score 70%, SD 14%) [44,45], 1 qualitative study (mean score 100%) [46], and 2 mixed method studies (mean score 60%, SD 0%) [47,48].

The lower quality of the quantitative descriptive studies was partly due to the lack of reporting on nonresponses in all studies. Similarly, the lower quality of the mixed methods studies stemmed from a lack of integration between qualitative and quantitative findings. For the 2 quantitative randomized controlled trials, the outcome assessors were not blinded, or the blinding process was not clearly reported.

### Characteristics of Included Studies

Overall, 4 [9,38,45,46] of the 18 selected studies were published in 2010, and the other 14 studies were published between 2021 and 2024. Around 12 studies used data collected between 2011 and 2023, including 4 studies [34,35,43,47] that explicitly addressed the effect of the COVID-19 pandemic. A total of 6 studies [37,39,41,44-46] did not state the year of data collection.

Regarding the geographic location, 2 studies [42,48] were conducted in Germany, 2 [9,35] in Japan, and 2 [40,47] in Italy, with the latter also analyzing a sample from the Netherlands. The remaining 12 studies were performed in Australia, Canada, China, Denmark, Israel, Latvia, Lebanon, Libya, Norway, Saudi Arabia, the United Kingdom, and the United States. Europe was the most represented region, with 8 studies, followed by 6 in Asia, 2 in North America, 1 in Oceania, and 1 in Africa.

Sample sizes ranged from 91 to 33,087, although 6 studies [36,39,40,43,45,46] did not report participant numbers. Research covered various economic sectors. Around 4 studies [32,36,41,46] focused on health sector–related occupations, including health care workers, home care service workers, radiation technologists, and radiation therapists. Most other studies examined occupations in sectors outside health care—particularly public institutions—or unspecified fields. These included upper-level nonmanual workers (eg, managers and professionals) [35], office, administrative, white collar, and public sector workers [37-40,45], production line employees [38], and computer users [44]. A Lebanese study examined self-employed individuals, freelancers, and temporary workers in any sector [43]. Overall, 1 [9] of 2 studies conducted in Japan focused on employees in electronics-related companies performing generally representative (not industry-specific) jobs, including administration, design, research and development, sales, and manufacturing. In total, 1 study [48] focused on farmers, gardeners, and foresters, while another included a mixed sample of health care and non–health care workers, students, and unemployed individuals [36]. Altogether, 4 studies [33,34,42,47] did not explicitly state respondents’ occupation or economic sector.

Regarding socioeconomic characteristics, age was analyzed differently across studies. A total of 6 studies [33,34,36,37,40,44] presented results for different age categories, while 2 studies [44,45] treated age as a continuous variable, 1 did both [35], and others studied 2 birth cohorts [42]. The remaining 7 studies [32,38,39,43,46-48] did not assess age differences, although they included employed participants aged 50 years and older. Only 3 studies [42,46,47] exclusively analyzed older workers, while all the others included both younger and older workers. Two studies [32,39] did not report gender, and 1 study [9] included both men and women but only analyzed men due to a low female sample size. The rest incorporated both sexes in their analyses. Regarding job position, only 5 studies [35,41,43,46,48] collected such data.

### Types of Digital Technologies Assessed

#### Work-Related Hardware and Communication Tools

Six studies explicitly investigated common workplace digital technologies. Three studies [9,36,37] studied the intensity of using computers, laptops, tablets, smartphones, mobile phones, emails, and websites. One [9] of those studies examined, besides occupational use, private use also (eg, gaming, email, and web browsing). Another study focused on video display units—covering mobile phones, laptops, computers, tablets, and electronic readers—in relation to computer vision syndrome (CVS) [40]. The other 2 studies [33,42] explored the impact of daily internet use on the physical health of older adults and older workers.

#### Teleworking

In 2 studies [35,43], digital technologies were not directly specified but were inferred, linking outcomes to remote or hybrid working arrangements where the role of digital technologies was implicit rather than explicitly described. One study [34] combined both perspectives by examining working from home alongside technical organizational support as a mediating factor

#### Workplace-Specific Technical Tools

Two studies examined technologies unique to specialized occupational settings: the presence and use of personal computers and picture archiving and communication systems among radiation technologists [41] and technical changes and adaptations in work processes, as identified through recommendations by mature radiation therapists in face-to-face interviews [46].

#### Digital Health and Support Interventions

The remaining 7 studies evaluated digital support programs and apps, namely (1) the Roczen Program, a medically-led, technology-enabled weight management initiative [39]; (2) digital support systems for managing neck pain [32]; (3) guided internet-based interventions, email communication, and electronic couch support (via telephone and internal messaging) for pain management [48]; (4) the AgeWell Digital Coach, a smartphone app with an avatar-based interface, motivational messaging, and activity tracking, designed to promote physical activity, mental well-being, and social engagement during the transition to retirement [47]; (5) an ergonomic learning program delivered digitally to promote healthier workplace practices [38]; (6) a novel “EYE ROLL” device connected to office computers to support vision relaxation [44]; and (7) a self-modeling webcam photos to reduce musculoskeletal risk among office workers using computers [45].

### Physical Health Assessment Instruments

#### Overview

To examine the physical health effects of digital technology use among employed (older) adults, various instruments were used across the selected studies, ranging from self-reported measures to clinical assessments.

#### Self-Reported Symptoms and Pain

Eight studies used self-reported questionnaires to assess physical symptoms associated with digital technology use. These included gathered self-reported ratings on headaches, back pain, neck pain, carpal tunnel syndrome, and CVS (ocular symptoms) [36] and chronic pain [48]. Neck pain intensity was assessed by asking participants whether they had been “bothered” by pain based on a previously published symptom checklist [32], and musculoskeletal pain or discomfort for 5 body regions was evaluated using response options ranging from never (0) to almost always (4) for frequency and mild (1) to severe (3) for severity [34]. Other studies used different scales, for example, a 0-10 point scale for musculoskeletal pain [38], an ergonomic survey [41], and the Rapid Upper Limb Assessment (RULA) to evaluate an innovative self-modeling photo training method for reducing musculoskeletal risk among office workers using computers [45]. Finally, 1 study [43] used a web-based cross-sectional self-report questionnaire with yes or no responses conducted via Google Forms on teleworking-related health problems among teleworkers to capture physical health outcomes (neck, shoulder, wrist, hand, and back pain), alongside chronic disease indicators (hypertension and diabetes).

#### Standardized Physical Health Surveys

Two studies [42,47] used the internationally validated SF-12 **(**12-Item Short-Form Health Survey) questionnaire to assess self-rated health status based on the 2 subscales, physical and mental health.

#### Computer Vision and Ocular Health

A qualitative study using an interpretive phenomenological approach conducted face-to-face interviews with radiation therapists aged more than 50 years to explore the ocular health effects of workplace technology [46]. In contrast, 4 quantitative studies assessed visual function and symptomatology using a range of clinical and self-reported measures. These include the application of the Computer Vision Syndrome-Q IT scale to capture the frequency and intensity of 16 visual symptoms, as well as tear stability (break-up time), corneal staining presence, and tear quantity using the Schirmer II test [40]. Other studies (1) collected self-reported symptom ratings, such as eye burning, itching, and eye pain, to calculate severity scores [36]; (2) conducted comprehensive vision examinations, including saccadic eye movement recordings and objective accommodation measurements [44]; and (3) measured ocular axial length (AL) of both eyes using an optical AL meter [9].

#### Clinical Biomarkers and Physiological Measures

One study evaluated the impact of a digitally enabled time-restricted eating intervention—The Roczen Program—by incorporating objective physiological measurements, including body weight (kg), BMI (kg/m²), waist circumference (cm), hemoglobin A_1c_ (HbA_1c_, mmol/mol), and blood pressure (BP); systolic and diastolic, mm Hg) [39]. Another study used objective and self-reported instruments to assess the impact of the indoor environment on individual well-being and physiological outcomes, including heart rate variability and BP. Physical activity levels were assessed by the ECGMove3’s triaxial accelerometer sensor [37].

#### Chronic Conditions and Cardiovascular Health

Two studies investigated the relationship between work and chronic health outcomes using self-reported data. One study [33] collected self-reported diagnoses of hypertension, arthritis, and asthma confirmed by medical professionals. Another study examined the association between cardiovascular risks and remote work, categorized into different levels of medical treatment and disease progression [35].

These diverse instruments highlight the multifaceted nature of physical health assessments in relation to digital technology use, encompassing subjective experiences, standardized scales, and clinical health indicators.

### Physical Health Domains

#### Overview

The studies on physical health outcomes included in the scoping review can be broadly grouped into 5 physical health domains: eye health, musculoskeletal health, metabolic and cardiovascular health, workplace sound levels, and user experiences of new digital technologies. Several studies span multiple categories. While some focus on the effects of work-related digital technology use on physical health, others evaluate interventions.

#### Eye Health

Eye health studies reported on visual strain, CVS, and eye discomfort. High rates of CVS symptoms, such as blurred vision, headaches, and burning sensations, were observed, particularly among workers using screens for more than 6 hours per day. Interventions like guided vision relaxation exercises showed promise in reducing symptoms. More specifically, 6 studies contained analysis regarding eye health–related issues. One study [36] reported a 38.6% prevalence of CVS among participants in Libya, identifying higher risk among individuals aged 45 years or older, students, and those using computers for more than 6 hours per day. Besides burning eyes, itching eyes, and increased sensitivity to light, other frequently reported physical health symptoms include headaches, back pain, neck pain, and shoulder pain. A study conducted among Italian office workers reported a higher prevalence of CVS (67.2%), with symptoms, including blurred vision (63.5%), perceived visual deterioration (61.8%), headaches (56.3%), and burning sensations in the eyes (54.2%). Although no significant age-related differences in CVS prevalence were found, women, individuals with intensive digital device use at work (>6 h/d) and those with optical correction were more affected [40]. In a study of male employees in Japanese electronics-related companies, use of work-related digital technology was associated with ocular AL elongation, especially among older workers [9]. Longitudinal data from China similarly suggested that regular internet use was associated with a lower risk of vision impairment [33]. A qualitative study involving medical radiation technologists aged 50 years and older revealed that many experienced deteriorating eyesight that negatively affected their work, especially when using progressive lenses, along with hearing loss [46]. Another study focused on remedies for eye problems connected with computer use by analyzing the effectiveness of an eye roll device designed for guided vision relaxation exercises in open workspaces. The intervention was found to alleviate eye-related complaints, such as eye fatigue and vision discomfort [44]. Although the quality score of included studies was low (mean score 63%, SD 29%), their findings consistently indicate that the duration and intensity of digital device use often have a greater impact on visual health than chronological age itself.

#### Musculoskeletal Health

Seven studies addressed musculoskeletal disorders, often associated with prolonged sitting or suboptimal workstation ergonomics. Neck, back, and shoulder pain were common, especially among teleworkers and those in health care roles. Ergonomic training programs, photo-based posture correction, and participatory interventions demonstrated efficacy in reducing musculoskeletal symptoms. One study [34] found that older workers who teleworked more than their preferred number of days had a higher likelihood of experiencing musculoskeletal pain. Another study identified back pain as a common drawback of telework among employees in Lebanon [43]. A study in Saudi Arabia focused on radiographic technologists and reported that 27.3% of participants experienced musculoskeletal complaints, with a higher prevalence among female and older workers. Contributing factors included the use of imaging equipment, particularly tasks involving patient transfer, wearing lead aprons, and moving heavy equipment [41]. A large-scale longitudinal study from China found that daily internet usage decreased the risk of hypertension, chronic lung disease, stroke, digestive disease, memory-related disorders, arthritis or rheumatism, asthma, depression, and vision impairment among both working and nonworking adults older than 45 years [33]. Three other studies [32,38,45] analyzed the effects of employers’ training on musculoskeletal disorders or symptom risks. According to a Norwegian study using data collected from home care service workers, the introduction of new technologies in the work environment, accompanied by user training, resulted in lower levels of neck pain reported by participants [32]. In a randomized controlled trial that compared 2 interventions: one based on conventional ergonomic training and workstation adjustment, and the other based on self-modeling photo training accompanied by conventional ergonomic training [45]. While both interventions had beneficial effects in improving work posture and reducing the risk of musculoskeletal disorders—particularly among older workers—only the intervention involving photo training has lasting effects, with stronger outcomes observed among women. In another study that evaluated the effects of a learning program focused on musculoskeletal health, specifically addressing low-strain working techniques, physical training, accompanied by participatory ergonomics, led to increased work productivity among participants [38]. Overall, the findings related to musculoskeletal health are derived from studies with an average quality score of 74% (SD 10%).

#### Metabolic and Cardiovascular Health

A total of 3 studies [33,35,39] dealt with chronic health conditions. A UK study assessed the impact of a health-related intervention implementing time‐restricted eating through the Roczen Program [39]. The authors found that after 1 year, study participants—most of whom were public sector workers—experienced weight loss and reductions in BMI and waist circumference. However, no statistically significant effects were observed for systolic and diastolic BP. A nationwide cohort study in Japan found no significantly increased risks of coronary heart disease among managers and professionals at older ages [35]. The authors concluded that while remote work (teleworking) elevates the risk for younger managers and professionals, it did not increase the risk for older counterparts. Finally, a Chinese study that analyzed 4 waves of the China Health and Retirement Longitudinal Study (CHARLS)—which included both working and nonworking participants aged 45 and older—found that daily internet use was associated with a lower incidence of several chronic conditions, including hypertension, arthritis, asthma, stroke, and vision impairment. However, the authors noted that these outcomes could be influenced by a sample selection effect, as healthier or more advantaged individuals are more likely to use the internet [33]. Overall positive outcomes of the aforementioned studies included reduced BMI and waist circumference through technology-enabled programs. Daily internet use was associated with decreased incidence of several chronic conditions, though some studies found no significant effects on BP or coronary heart disease risk among older professionals. Despite the few identified studies on metabolic and cardiovascular health, their average quality score was relatively high (mean score 87%, SD 12%).

#### Workplace Environment and Sound

Three studies examined workplace sound levels. One study [37] analyzed the effects of sound levels on public institution workers in the United States, finding that sound levels at or above 50 decibels (A-weighted) increased variability in anxiety levels. Contributing factors to interpersonal differences in the sound–well-being association included age, BMI, high BP, and intensive computer use. Supporting this finding, a Canadian study offered a qualitative perspective, in which a radiation technologist expressed frustration over constant workplace noise and its negative impact on the work environment [46]. In another study addressing workplace health risks, digital technology use did not negatively impact workability or physical health across different occupational sectors in Germany, provided that the use was not perceived as digital work intensification [42]. Overall, elevated noise levels were linked to increased physiological stress, particularly in office settings with dense computer use. Participant narratives also revealed frustration related to environmental noise. The mean quality score of the 3 studies was 93% (SD 12%).

#### User Experience and Engagement

Finally, 2 studies focused on users’ perspectives, barriers, and facilitators rather than direct health outcomes of digital technology use among older workers. One specifically on the design and user experience of a virtual coaching app to support healthy aging among older workers approaching retirement. Findings suggested the need to enhance physical activity and self-efficacy, and increase emotional and informational support during the transition to retirement [47]. The other study highlighted engagement challenges and user perceptions of internet-based interventions (IBIs) designed to prevent depression and manage pain-related disability in green professions, such as farming, gardening, and forestry. Key barriers identified included mental health stigma (especially in rural settings), low prioritization of mental well-being compared to physical health, financial stress, and the physically demanding nature of agricultural work. Only 22.2% of participants completed at least 80% of intervention modules, with farmers facing greater challenges due to the work-related pain and financial strain. However, the autonomy and confidentiality of IBIs appeal to rural users, and peer or family support enhances participation. Thus, IBIs hold potential, addressing engagement barriers crucial for effectiveness [48]. Nevertheless, it should be noted that the results from these 2 intervention studies should be cautiously approached due to their moderate quality score of 60% (SD 0%).

## Discussion

### Addressing a Research Gap: Physical Health in the Digital Workplace

The increasing integration of digital technologies into daily life and work environments has raised the need for researchers to examine their impact on the physical health of older workers. While previous scoping and systematic reviews have predominantly focused on psychosocial outcomes, such as technostress and burnout [27], evidence on physical health consequences has remained fragmented. This scoping review addresses that gap by mapping the physical health impacts of digital technologies among older workers across diverse occupational sectors. Our synthesis shows that digitalization has multifaceted effects on eye health, musculoskeletal health, metabolic and cardiovascular health, and workability, with risks and benefits often varying by age, context, and work design. The integration of digital technologies into the workplace also presents both opportunities and risks. On the one hand, digital tools can automate physically demanding tasks, optimizing work processes and thereby reducing physical strain for older workers [51,52]. However, despite the frequent use of technologies like smartphones, laptops, and tablets, comprehensive studies directly linking digital work environments to physical health outcomes among older workers remain scarce. This highlights critical gaps in the evidence base—including the need for longitudinal designs, sector-specific analyses, and user-centered interventions—that should guide future research. In this way, the review contributes to an expanding body of evidence aiming to ensure that the digital transformation of work supports rather than undermines healthy aging.

### Thematic Insights Into Physical Health Domains

This review highlights several key domains in which digital technologies affect physical health outcomes:

1. Eye health: Multiple studies reported a heightened prevalence of CVS in older workers exposed to prolonged screen use. Risk factors included longer exposure duration, older age, female gender, and pre-existing optical correction needs. Importantly, interventions, such as guided vision relaxation exercises, demonstrated efficacy, indicating that workplace-based preventive strategies can mitigate visual strain.
2. Musculoskeletal health: Increased technology use linked to remote and hybrid work arrangements was consistently associated with musculoskeletal complaints (neck, back, and shoulder pain). Evidence supports ergonomic interventions, such as participatory training and workstation modification, as effective countermeasures. These findings confirm earlier work on sedentary behavior but extend it by situating musculoskeletal risks specifically within digitalized work environments.
3. Metabolic and cardiovascular health: Evidence in this domain was more mixed. While teleworking was associated with elevated coronary heart disease risk among younger professionals, no such association was observed in older workers. Conversely, daily internet use appeared protective, reducing the likelihood of chronic disease among adults aged 45 years and older. These findings suggest that digital engagement may have differential effects across age groups, pointing to the need for more nuanced, longitudinal research.
4. Workability and career transitions: digital interventions, such as virtual coaching apps and internet-based support systems, demonstrated promise in enhancing physical activity, self-efficacy, and emotional well-being during key career transitions, including preparation for retirement. When tailored to the needs of older workers, these tools were generally well received. However, barriers to engagement, such as low digital literacy, rural isolation, and physical fatigue, particularly in physically demanding sectors, were frequently noted. These challenges highlight the importance of cultural tailoring, digital literacy support, and user-centered design in the development of effective workplace digital interventions.

Together, these thematic insights suggest that digitalization in later working life is neither uniformly beneficial nor uniformly harmful but rather context-dependent, shaped by technology type, work design, and worker characteristics.

### Designing Digital Interventions for Healthy Aging at Work

Based on our findings, we emphasize the critical role of employers in mitigating the physical health risks associated with digital work environments. Organizations should proactively implement measures to safeguard the physical well-being of older employees in digitalized work settings. Potential strategies include the following:

1. Adaptive ergonomic workstations with adjustable desks, chairs, and screen positions to meet the health needs of aging workers, specifically related to musculoskeletal health and visual functions.
2. Wearable health devices that monitor posture, track movement, and screen time to provide real-time feedback and encourage regular breaks and stretching exercises.
3. Hiring of occupational health specialists to assess individual health risks and customize intervention strategies for older workers.
4. Employers to support physical activity either by introducing active-break routines at work or by arranging external events like weekend activities and retreats, which focus on physical exercises to benefit musculoskeletal health.
5. Workplace wellness programs, such as (virtual) physiotherapy, guided stretching sessions, and eye health workshops, can help to prevent musculoskeletal and digital eye strain.

Employers who integrate technology with human-centered design principles can create digital workspaces that promote both the health and inclusion of older employees, fostering a sustainable and age-inclusive workforce.

### Limitations

A potential limitation of this scoping review is that, despite an extensive and systematic search across 7 major academic databases, some relevant studies may have been missed. Non-English language publications were excluded due to resource constraints, which may have introduced language bias. Given the rapid pace of digital innovation, newer studies may also have been published after our search was completed. Our analysis reflects only the studies retrieved through our search strategy, with most conducted in Europe and Asia. Therefore, the findings are more applicable to these regions, and future studies from underrepresented parts of the world are needed for a more global understanding. Moreover, cultural and regional workplace differences may influence the applicability of findings. Occupational health frameworks, ergonomic standards, digital literacy, and cultural attitudes toward aging and technology vary across regions, meaning that experiences of digital work in, for example, Norway or Japan may not be directly comparable to contexts with fewer resources or less employer support. These contextual differences highlight the need for future studies to examine workplace cultures and policy environments when assessing the transferability of interventions.

Another limitation concerns the varying definitions of digital technology. Some studies examined explicit tools (eg, personal computers, mobile devices, and specialized apps), while others focused on implicit contexts (eg, telework) where technology use was assumed but not specified. By including both types, the breadth of digitalization in the workplace was captured, but this variability may limit the comparability of findings across studies.

Regarding study design, most intervention-based studies (including 8 of the 9 quantitative nonrandomized, both quantitative randomized controlled trials, and both mixed method studies) were limited in exposure duration, ranging from just 6 weeks to 2.5 years, while the remaining studies were primarily cross-sectional or descriptive.

Our review also highlights broader methodological limitations in the current literature. Many studies rely on cross-sectional designs, restricting the ability to establish causal relationships between technology use and physical health outcomes. A few exceptions point to promising directions; for instance, 1 study [37] used repeated measures and found that both low and high sound levels negatively affected physiological well-being. Such findings suggest that workplace designs addressing sensory environments—for example, providing quiet workspaces for older employees—may represent an important avenue for improving health.

Future studies should therefore emphasize longer-term, longitudinal, and workplace-based intervention designs to better capture the sustained impact of digital technology use on the physical health of older workers.

While our review identifies several ergonomic and digital health programs that may benefit older workers’ physical health [eg, 37,43,44]—demonstrating both the feasibility and potential benefits of workplace-based strategies—the strength of evidence is limited by small samples, heterogeneous designs, and short-term effects. As with all scoping reviews, our goal was to map the existing literature rather than formally evaluate intervention effectiveness. To strengthen our approach, we followed the PRISMA-ScR checklist (Multimedia Appendix 4) and critically appraised all included studies using the MMAT, which allowed us to assess the methodological quality of both quantitative and qualitative evidence and provide a more informed synthesis (Multimedia Appendix 3). Looking ahead, systematic reviews or meta-analyses should apply formal evidence grading frameworks to more rigorously assess the robustness of specific interventions and inform workplace policy.

### Conclusions

To the best of our knowledge, this scoping review is the first to provide a comprehensive synthesis of the physical health effects associated with digital technologies in the workplace, offering valuable insights into how digitalization influences the well-being of older workers. The findings highlight a diverse range of physical health implications, spanning eye health, musculoskeletal conditions, metabolic and cardiovascular health, and workplace ergonomics. These results underscore the pressing need for targeted workplace interventions and employer-driven initiatives to ensure a healthier work environment for aging employees.

Although intervention studies remain scarce, existing examples—such as ergonomic learning programs, vision relaxation exercises, and posture improvement interventions—suggest promising avenues. Future research should build on these findings by developing and evaluating evidence-based interventional programs tailored to older workers and diverse workplace contexts, while also conducting longitudinal studies to examine causal relationships between digital technology use and physical health outcomes in greater detail.

Additionally, the design and implementation of digital technologies should adopt an inclusive and adaptive approach, acknowledging age-related changes in sensory, cognitive, and physical functions. Participatory design models—in which older workers actively contribute to the development of digital workplace solutions—will be critical for ensuring usability, acceptance, and sustained engagement.

In conclusion, this review reinforces the necessity of adopting a holistic and integrated approach to safeguarding the physical health of older workers in an increasingly digitalized labor market. By addressing the physical, ergonomic, and psychosocial challenges of modern work environments and leveraging adaptive technologies, it is possible to support healthy aging at work, extend career longevity, and maintain the well-being and productivity of older employees in a sustainable manner.

## Appendix

Multimedia Appendix 1 Definitions of the inclusion criteria regarding population, concept, and context in the scoping review.

Multimedia Appendix 2 Description of studies selected (N = 18).

Multimedia Appendix 3 Qualitative assessment (Mixed Methods Appraisal Tool) of selected studies (N = 18).

Multimedia Appendix 4 PRISMA-ScR checklist.

## Abbreviations

AL: axial length
BP: blood pressure
CHARLS: China Health and Retirement Longitudinal Study
EU: European Union
HbA1c: hemoglobin A1c
IBIs: internet-based interventions
CVS: computer vision syndrome
IRRID: International Registered Report Identifier
MMAT: Mixed Method Appraisal Tool
PCC: population, concepts, and context
PRISMA: Preferred Reporting Items for Systematic Reviews and Meta-Analyses
PRISMA-ScR: Preferred Reporting Items for Systematic Reviews and Meta-Analyses extension for Scoping Reviews
RULA: Rapid Upper Limb Assessment
SF-12: 12-Item Short-Form Health Survey
