# Supplementary material for: The Impact of Digital Technology on the Physical Health of Older Workers: Scoping Review
Source: JMIR Aging. 2025 Nov 18;8:e78406. doi: 10.2196/78406 (PMC12673309; doi:10.2196/78406)
Supplement: Multimedia Appendix 1 [file aging_v8i1e78406_app1.pdf]

Multimedia Appendix 1. Definitions of the inclusion criteria regarding PCC in the scoping review.

Population: older workers

- Older workers include study participants employed at the moment of the study
- Study participants must be aged 50 years or older. When only age ranges are analysed, the age of 50 should be included in the youngest age category (eg, 45-54). While there are ongoing debates regarding what age defines older workers [29], we have opted to include individuals 50 years or older. This decision is based on the increasing presence of this cohort in the labor market, their likelihood of remaining in the workforce longer than previous generations, and the need to recognize the diversity within this demographic group. People in their 50s may have varying career trajectories, skill sets, and motivations for remaining in the workforce. By defining older workers as those aged 50 and over, organisations can more effectively cater to the unique needs and experiences of this diverse and increasing group of older individuals in the labor market.
- If age is treated as a continuous variable rather than analysed as categories, the sample also has to have participants aged 50 years or older, i.e. younger ages are only permitted if older ages are also represented in the study.

Concept: digital technologies

- Digital technologies refer to data manipulation, storage, transmission, and processing in binary data [30]. It allows for the interaction with stored data using electronic devices (eg, computers, microprocessors). Digital data can be stored in various digital storage media (eg, hard drives, solid-state drives, memory cards and cloud storage). Furthermore, digital technology also enables the transmission of data over digital communication networks
(eg, the internet, local area networks, wireless networks).
- Digital technologies are defined as any type of digital tool or device used in the context of (creative) production, (ie, studies or study results that look at the effect of digital technologies not related to work, eg, for health management, are excluded).
- Digital technologies include the use of computers at home for work (e.g. teleworking) as well as more recent digital technologies or tools (eg, apps) but only if they are used for work purposes.
- Studies will be excluded if working from home does not involve digital tools (eg, only landlines are used).

Context: physical health

- Any physical health outcome is accepted.
- Mental health outcomes are excluded unless mentioned in combination with a physical health outcome.
- Studies will be excluded if the health outcome is not associated with an effect of the use of digital technologies in the work sphere.

References

29. McCarthy J, Heraty N, Cross C, Cleveland JN. Who is considered an ‘older worker'? Extending our conceptualisation of ‘older’from an organisational decision maker perspective. Human Resource Management Journal. 2014;24(4):374-393 [doi: 10.1111/1748-8583.12041]
30. LaMeres BJ. Introduction: analog vs. digital. In: LaMeres BJ, ed. Introduction to Logic Circuits & Logic Design with VHDL. Cham: Springer; 2017:1-5 [doi: 10.1007/978-3-319-53883-9_1]
