# Supplementary material for: The Impact of Digital Technology on the Physical Health of Older Workers: Scoping Review
Source: JMIR Aging. 2025 Nov 18;8:e78406. doi: 10.2196/78406 (PMC12673309; doi:10.2196/78406)
Supplement: Multimedia Appendix 2 [file aging_v8i1e78406_app2.pdf]

Multimedia Appendix 2. Description of studies selected (*N* = 18)

Author (year) [reference in article]	Country	Year of data collection	N (invited/ participants/ follow-ups	Study design	Age used in the analysis /results[a]	Young & older workers/ older adults & workers	Gender[b]	Population	Type and specific digital tool (DT)	Physical health assessment instruments	Physical health outcomes
Alturaiki et al. (2023) [36]	LY	2023	NS/470/NA	Quantitative non-randomized (cross-sectional) study	AC: <25, 25-45, >45	Yes/Yes	F and M	health care sector; non health care sector; students; unemployed	Explicit (computer, tablet, smartphone, TV)	CVS, both ocular (e.g. burning eyes, itching eyes, eye pain) and non-ocular symptoms (headache, back pain, neck pain)	Higher risk of CSV among individuals aged 45 years or older and those using computers for more than six hours per day; back pain as a common drawback of telework
Borle et al. (2021) [42]	DE	2018	3180/3133/NA	Quantitative des-criptive (survey-based cross-sectional study)	Cohort: 1959 and 1965	No/No	F and M	sector representing Germany's socially insured workforce.	Explicit (ICT use (PC, laptop, mobile phone, email, websites, etc) and digital work intensification).	Self-reported physical health	No impact of digital technology use on workability or physical health across different occupational sectors
Braun et al. (2022) [48]	DE	2019	Sample PROD-A 360/161/interview 22<br>Sample PACT-A 256/39/interview 19.<br>Follow up 8/30.	Mixed-method study	Mean (SD): 55.88 (7.86)	Yes/Yes	F and M	Green professions (farmers, gardeners, and foresters)	Explicit (internet, web-based assessment, emails, e-couch support via telephone or internal messageing function)	Chronic pain (PACT-A sample)	Work-related pain and financial strain as perceived barriers for the use of internet-based interventions (IBIs) designed to prevent depression and manage pain-related disability in green professions such as farming, gardening, and forestry
Brown et. al (2024) [39]	UK	not stated	NS/baseline 660/<br>3 months 316/<br>6 months 204/<br>9months 137/<br>12 months 82	Quantitative non-randomized (retrospective observational) study	Mean (SD): 47.5 (10.1). Age used as control var.	Yes/Yes	T	public sector workers (89%)	Explicit (Roczen Program, a medically-led, tech-enabled weight management program aimed to help improve metabolic health, quality of life, and reduce disease risk).	Bodyweight, BMI, waist circumference, Systolic blood pressure, Diastolic blood pressure	Positive effect of a health-related intervention implementing time-restricted eating through the Roczen Program on weight loss and reductions in BMI and waist circumference among public sector workers, but no impact of the intervention on systolic and diastolic blood pressure
Cantó-Sancho et al. (2023) [40]	IT	2019	NS/238/NA	Quantitative descriptive (prevalence) study	AC: < 40, 40+	Yes/No	F and M	hospital employees	Explicit (Video Display Units at work: mobile phones, laptops, computers, tablets, or e-readers).	CVS, tear stability	No significant age-related differences in CVS prevalence, higher risk of CSV among individuals with intensive digital device use at work (>6 hours/day)
Christensen & Johannessen (2024) [32]	NO	2019	6997/1885/887	Quantitative non-randomized (prospective) study)	mean age (SD)=45 (11.6). Age used as a control var.	Yes/No	T	health care-home care service workers	Explicit (digital support systems)	Neck pain	Lower levels of neck pain reported by home care service workers as a result of the introduction of new technologies in the work environment
Haddad et al. (2024) [43]	LB	2022-23	NS/230/NA	Quantitative descriptive	mean age (SD)=30.55 (10.80).	Yes/No	F and M	not stated	Implicit (working from home (no	Musculoskeletal problems (neck, shoulder, wrist, hand,	Back pain as a common drawback of telework among employees

				(survey) study)	Age used as a control var.				digital tool specified))	etc.), back pain, chronic diseases (hypertension, high cholesterol, diabetes, etc.), visual problems	
Honda et al (2019) [9]	JP	2015-17	8989/8606 (valid cases)/NA	Quantitative non-randomized (cross-sectional) study	AC: 35-40, 41-45, 46-50, 51-55, 56-60, 61-65	Yes/No	M (F not analysed)	Employed in electronics-related companies	Explicit (work-related and private ICT use[d])	Ocular axial length (AL) elongation	Positive association between work-related digital technology use and ocular AL elongation among older workers
Li et al. (2023) [33]	CN	2011-18 (4 waves)	25589/20113 longitudinal 17117 cross-sec/NS	Quantitative non-randomized (cohort) study	AC: 45–54, 55–64, 65–74, 75+	Yes/Yes	F and M	not stated, incl. both working, not working/retired	Explicit (daily internet use)	14 noncommunicable chronic diseases[c] and vision impairment	Association between regular internet use and decreased risk of hypertension, chronic lung disease, stroke, digestive disease, memory-related disorders, arthritis or rheumatism, asthma, depression, and vision impairment among both working and nonworking adults over the age of 45
Oakman et al. (2023) [34]	AU	2021-22	wave1 637/451/NA wave2 657/358/NA wave3 669/320/NA	Quantitative non-randomized (cohort) study	AC: <46, 46+	Yes/No	F and M	not stated	Explicit and implicit (working from home and technical support from organization (mediator))	Musculoskeletal pain	The positive association between exceeding preferred telework days and higher musculoskeletal pain incidence among older workers
Santini et al., (2023) [47]	IT and NL	2021	91/62/T1 52/T2 49	Mixed-method study	AR: 55+	No/Yes	F and M	(former) employees 3 years before or 3 years after retirement	Explicit (AgeWell Digital Coach (a smartphone app, incl. features such as an avatar-based interface, motivational messages, and activity tracking)).	Perceived physical health and physical activity	Positive experience of older workers approaching retirement with the use of a Virtual Coaching application to support healthy ageing
Sell et al. (2016) [38]	DK	2006-09	Baseline 538/448 1st follow-up 478/637 2nd follow-up 428/617. From baseline to 2nd follow-up: 249	Quantitative non-randomized (quasi-experimental) study	Mean (SD): 43.9 (9.2)	Yes/No	F and M	not stated	Explicit (ergonomic learning program)	Musculoskeletal pain in neck, shoulders, elbows, hands, back, lower back, hips, knees and feet	Positive effects of a learning program focused on musculoskeletal health on work productivity
Shubayr & Alashaban (2022) [41]	SA	not stated	800/381/NA	Quantitative descriptive (prevalence) study	AR: 20-29, 30-39, 40-49, 50-59	Yes/No	F and M	workers in public and private hospitals	Explicit (personal Computer/Picture Archiving and Communication System (PACS))	Musculoskeletal symptoms (work-related symptoms of pain, stiffness, soreness, cramping in either extremities, neck or back areas)	Higher prevalence of musculoskeletal complaints among female and older radiographic technologists

Srinivasan et al. (2023) [37]	US	not stated	248/231	Quantitative non-randomized (cross - sectional) study with repeated measures over three days	AC: <30, 30–40, 40–50, 50–60, 60+	Yes/No	F and M	office workers in public institutions	Explicit (intensity of computer use)	The effect of sound levels on physiological well-being, including BMI and high blood pressure.	Positive association between sound levels at or above 50 decibels (A-weighted) and increased variability in anxiety levels among public institution workers, with the impact varying depending on their age, BMI, high BP, and the intensity of computer use
Svede et al (2024) [44]	LV	not stated	590/126/89	Quantitative randomized control trial	AR: 17-50 (4 older than 40). Used as continuous variable.	Yes/No	F and M	not stated	Explicit (a novel EYE ROLL device designed to facilitate guided vision relaxation exercises in an open space, connected with the office computer).	Asthenopic complaints and visual functions, saccadic eye movements, accommodation response (CVS)	Eye roll device for guided vision relaxation being effective in reducing eye fatigue and visual discomfort in open workspaces
Taieb-Maimon et al. (2012) [45]	IL	not stated	NS/60/60	Quantitative randomized control trial	AR: 23-66, analyzed as continuous variable.	Yes/No	F and M	workers in research university and university hospital	Explicit (self-modeling webcam photos)	Low back symptoms, neck pain, musculoskeletal discomfort, sitting posture	Beneficial effects of self-modeling photo training accompanied by conventional ergonomic training in improving work posture and reducing the risk of musculoskeletal disorders
Wilson (2014) [46]	CA	not stated	NS/8/NA	Qualitative study	AR: 50+	No/No	F and M	health care	Explicit (technical changes)	Aches and pains, eyesight issues, hearing loss, meno-pause-related symptoms	Experience of eyesight deterioration and hair loss in medical radiation technologists aged 50 years and older, perceived frustration over constant workplace noise
Zaitsu et al. (2024) [35]	JP	2020-21	33087/27036 (valid cases) /18560 (valid cases)	Quantitative non-randomized (cohort) study	AC: 20–49, 50–65, and as continuous variable	Yes/No	F and M	not stated	Implicit (working from home)	Coronary heart disease (CHD) (angina pectoris/myocardial infarction) incidence	In contrast to younger managers, no increased risks of coronary heart disease among managers and professionals at older ages due to remote work

Notes: NA = not applicable; NS = not stated; DT = digital tool. CVS = Computer vision syndrome. ICT = Information and Communication Technology; LY = Libya, DE = Germany, UK = United Kingdom, IT = Italy, NO = Norway, LB = Lebanon, JP = Japan, CN = China, AU = Australie, NL = Netherlands, DK = Denmark, SA = Saudi Arabia, US = United States, LV = Latvia, IL = Israel, CA = Canada.

[a]Age is reported: AC = age categories, AR = age ranges or MEAN = mean age and SD = standard deviations.

[b]Gender is reported as categories used in the analysis or results: F = female, M = male, T = total (sex not specified).

[c]Hypertension, dyslipidemia, diabetes, cancer, chronic lung disease, liver disease, heart disease, stroke, kidney disease, digestive disease, emotional and psychiatric disease, memory-related disorders, arthritis or rheumatism, and asthma

[d]Work-related: computer-aided design and computer-aided manufacturing, programming, operator job, word processing, designer, assembling presentation materials, sending e-mails, and browsing websites. Private use: playing a game, sending e-mail, and browsing web sites.
