# Supplementary material for: The Impact of Digital Technology on the Physical Health of Older Workers: Scoping Review
Source: JMIR Aging. 2025 Nov 18;8:e78406. doi: 10.2196/78406 (PMC12673309; doi:10.2196/78406)
Supplement: Multimedia Appendix 3 [file aging_v8i1e78406_app3.pdf]

Multimedia Appendix 3. Qualitative assessment (Mixed Methods Appraisal Tool) of selected studies (*N* = 18)

1. Quantitative non-randomized studies

**Author(s) and reference # in article**	**Screening questions (S) and methodological quality criteria**	**Responses***		
		**Yes**	**No**	**Can't tell**
1. Alturaiki et al. (2023) [36]	S1. Are there clear research questions?	1		
	S2. Do the collected data allow to address the research questions?	1		
	1.1. Are the participants representative of the target population?			X
	1.2. Are measurements appropriate regarding both the outcome and exposure/intervention?	0.2		
	1.3. Are there complete outcome data?			X
	1.4. Are the confounders accounted for in the design and analysis?			X
	1.5 During the study period, did the exposure/intervention occur as intended?	0.2		
	**Score methodological quality criteria**	**40%**		
2. Brown et al. (2024) [39]	S1. Are there clear research questions?	1		
	S2. Do the collected data allow to address the research questions?	1		
	1.1. Are the participants representative of the target population?	0.2		
	1.2. Are measurements appropriate regarding both the outcome and exposure/intervention?	0.2		
	1.3. Are there complete outcome data?			X
	1.4. Are the confounders accounted for in the design and analysis?	0.2		
	1.5 During the study period, did the exposure/intervention occur as intended?	0.2		
	**Score methodological quality criteria**	**80%**		
3. Christensen et al. (2024) [32]	S1. Are there clear research questions?	1		
	S2. Do the collected data allow to address the research questions?	1		
	1.1. Are the participants representative of the target population?	0.2		
	1.2. Are measurements appropriate regarding both the outcome and exposure/intervention?	0.2		
	1.3. Are there complete outcome data?			X
	1.4. Are the confounders accounted for in the design and analysis?	0.2		
	1.5 During the study period, did the exposure/intervention occur as intended?	0.2		
	**Score methodological quality criteria**	**80%**		
4. Honda et al. (2019) [9]	S1. Are there clear research questions?	1		
	S2. Do the collected data allow to address the research questions?	1		
	1.1. Are the participants representative of the target population?			X
	1.2. Are measurements appropriate regarding both the outcome and exposure/intervention?	0.2		
	1.3. Are there complete outcome data?	0.2		
	1.4. Are the confounders accounted for in the design and analysis?	0.2		
	1.5 During the study period, did the exposure/intervention occur as intended?	0.2		
	**Score methodological quality criteria**	**80%**		
5. Li et al. (2023) [33]	S1. Are there clear research questions?	1		
	S2. Do the collected data allow to address the research questions?	1		
	1.1. Are the participants representative of the target population?	0.2		
	1.2. Are measurements appropriate regarding both the outcome and exposure/intervention?	0.2		
	1.3. Are there complete outcome data?	0.2		
	1.4. Are the confounders accounted for in the design and analysis?	0.2		
	1.5 During the study period, did the exposure/intervention occur as intended?			X
	**Score methodological quality criteria**	**80%**		
6. Oakman et al. (2023) [34]	S1. Are there clear research questions?	1		
	S2. Do the collected data allow to address the research questions?	1		
	1.1. Are the participants representative of the target population?			X
	1.2. Are measurements appropriate regarding both the outcome and exposure/intervention?	0.2		
	1.3. Are there complete outcome data?			X
	1.4. Are the confounders accounted for in the design and analysis?	0.2		
	1.5 During the study period, did the exposure/intervention occur as intended?	0.2		
	**Score methodological quality criteria**	**60%**		
7. Sell et al. (2016) [38]	S1. Are there clear research questions?	1		
	S2. Do the collected data allow to address the research questions?	1		
	1.1. Are the participants representative of the target population?	0.2		
	1.2. Are measurements appropriate regarding both the outcome and exposure/intervention?	0.2		

	1.3. Are there complete outcome data?			X
	1.4. Are the confounders accounted for in the design and analysis?			X
	1.5 During the study period, did the exposure/intervention occur as intended?	0.2		
	**Score methodological quality criteria**	**60%**		
8. Srinivasan et al. (2023) [37]	S1. Are there clear research questions?	1		
	S2. Do the collected data allow to address the research questions?	1		
	1.1. Are the participants representative of the target population?	0.2		
	1.2. Are measurements appropriate regarding both the outcome and exposure/intervention?	0.2		
	1.3. Are there complete outcome data?	0.2		
	1.4. Are the confounders accounted for in the design and analysis?	0.2		
	1.5 During the study period, did the exposure/intervention occur as intended?	0.2		
	**Score methodological quality criteria**	**100%**		
9. Zaitsu et al. (2024) [35]	S1. Are there clear research questions?	1		
	S2. Do the collected data allow to address the research questions?	1		
	1.1. Are the participants representative of the target population?	0.2		
	1.2. Are measurements appropriate regarding both the outcome and exposure/intervention?	0.2		
	1.3. Are there complete outcome data?	0.2		
	1.4. Are the confounders accounted for in the design and analysis?	0.2		
	1.5 During the study period, did the exposure/intervention occur as intended?	0.2		
	**Score methodological quality criteria**	**100%**		
**Mean score of all studies**	S1. Are there clear research questions?	1		
	S2. Do the collected data allow to address the research questions?	1		
	1.1. Are the participants representative of the target population?	0.20		
	1.2. Are measurements appropriate regarding both the outcome and exposure/intervention?	0.13		
	1.3. Are there complete outcome data?	0.09		
	1.4. Are the confounders accounted for in the design and analysis?	0.16		
	1.5 During the study period, did the exposure/intervention occur as intended?	0.18		
	**Score methodological quality criteria**	**76%**		

* Supporting comments can be obtained from corresponding author.

2. Qualitative descriptive

**Author(s)**	**Screening questions (S) and methodological quality criteria**	**Responses***		
		**Yes**	**No**	**Can't tell**
1. Borle et al. (2021) [42]	S1. Are there clear research questions?	1		
	S2. Do the collected data allow to address the research questions?	1		
	2.1. Is the sampling strategy relevant to address the research question?	0.2		
	2.2. Is the sample representative of the target population?	0.2		
	2.3. Are the measurements appropriate?	0.2		
	2.4. How many participants did not answer/attrition for follow-up (%)		X	
	2.5 Is the statistical analysis appropriate to answer the research question?	0.2		
	**Score methodological quality criteria**	**80%**		
2. Cantó-Sancho et al. (2023) [40]	S1. Are there clear research questions?	1		
	S2. Do the collected data allow to address the research questions?	1		
	2.1. Is the sampling strategy relevant to address the research question?			X
	2.2. Is the sample representative of the target population?	0.2		
	2.3. Are the measurements appropriate?		X	
	2.4. How many participants did not answer/attrition for follow-up (%)			X
	2.5 Is the statistical analysis appropriate to answer the research question?		X	
	**Score methodological quality criteria**	**20%**		
3. Haddad et al. (2024) [43]	S1. Are there clear research questions?	1		
	S2. Do the collected data allow to address the research questions?	1		
	2.1. Is the sampling strategy relevant to address the research question?	0.2		
	2.2. Is the sample representative of the target population?	0.2		
	2.3. Are the measurements appropriate?	0.2		
	2.4. How many participants did not answer/attrition for follow-up (%)			X
	2.5 Is the statistical analysis appropriate to answer the research question?	0.2		
	**Score methodological quality criteria**	**80%**		
4. Shubayr and Alashban (2022) [41]	S1. Are there clear research questions?	1		
	S2. Do the collected data allow to address the research questions?	1		
	2.1. Is the sampling strategy relevant to address the research question?	0.2		
	2.2. Is the sample representative of the target population?	0.2		
	2.3. Are the measurements appropriate?	0.2		
	2.4. How many participants did not answer/attrition for follow-up (%)		X	
	2.5 Is the statistical analysis appropriate to answer the research question?	0.2		
	**Score methodological quality criteria**	**80%**		
**Mean score of all studies**	S1. Are there clear research questions?	1		
	S2. Do the collected data allow to address the research questions?	1		
	2.1. Is the sampling strategy relevant to address the research question?	0.15		
	2.2. Is the sample representative of the target population?	0.20		
	2.3. Are the measurements appropriate?	0.15		
	2.4. How many participants did not answer/attrition for follow-up (%)	0.00		
	2.5 Is the statistical analysis appropriate to answer the research question?	0.15		
	**Score methodological quality criteria**	**65%**		

* Supporting comments can be obtained from corresponding author.

3. Quantitative randomized control trial

Author(s)	Screening questions (S) and methodological quality criteria	Responses*		
		**Yes**	**No**	**Can't tell**
1. Svede et al. (2024) [44]	S1. Are there clear research questions?	1		
	S2. Do the collected data allow to address the research questions?	1		
	3.1. Is randomisation appropriately performed?	0.2		
	3.2. Are the groups comparable over time?	0.2		
	3.3. Are there complete outcome data?			X
	3.4. Are outcome assessors blinded to the intervention provided?			X
	3.5 Did the participants adhere to the assigned intervention?	0.2		
	**Score methodological quality criteria**	**60%**		
2. Taieb-Maimon et al. (2012) [45]	S1. Are there clear research questions?	1		
	S2. Do the collected data allow to address the research questions?	1		
	3.1. Is randomisation appropriately performed?	0.2		
	3.2. Are the groups comparable over time?	0.2		
	3.3. Are there complete outcome data?	0.2		
	3.4. Are outcome assessors blinded to the intervention provided?		X	
	3.5 Did the participants adhere to the assigned intervention?	0.2		
	**Score methodological quality criteria**	**80%**		
**Mean score of all studies**	S1. Are there clear research questions?	1		
	S2. Do the collected data allow to address the research questions?	1		
	3.1. Is randomisation appropriately performed?	0.20		
	3.2. Are the groups comparable over time?	0.20		
	3.3. Are there complete outcome data?	0.10		
	3.4. Are outcome assessors blinded to the intervention provided?	0.00		
	3.5 Did the participants adhere to the assigned intervention?	0.20		
	**Score methodological quality criteria**	**70%**		

* Supporting comments can be obtained from corresponding author.

4. Qualitative

Author(s)	Screening questions (S) and methodological quality criteria	Responses*		
		**Yes**	**No**	**Can't tell**
1. Wilson (2014) [46]	S1. Are there clear research questions?	1		
	S2. Do the collected data allow to address the research questions?	1		
	4.1. Is the qualitative approach appropriate to answer the research question?	0.2		
	4.2. Are the qualitative data collection methods adequate to address the research question?	0.2		
	4.3. Are the findings adequately derived from the data?	0.2		
	4.4. Is the interpretation of results sufficiently substantiated by data?	0.2		
	4.5 Is there coherence between qualitative data sources, collection, analysis and interpretation?	0.2		
	**Score methodological quality criteria**	**100%**		

* Supporting comments can be obtained from corresponding author.

5. Mixed methods

Author(s)	Screening questions (S) and methodological quality criteria	Responses*		
		Yes	No	Can't tell
1. Braun et al. (2022) [48]	S1. Are there clear research questions?	1		
	S2. Do the collected data allow to address the research questions?	1		
	5.1. Is rationale adequate for using a mixed methods design to address research question?	0.2		
	5.2. Are study components effectively integrated to answer the research question?	0.2		
	5.3. Are outputs of integration of qualitative & quantitative components adequately interpreted?			X
	5.4. Are divergences & inconsistencies between quantitative & qualitative results adequately addressed?			X
	5.5 Do the different study components adhere to the quality criteria of each tradition of the methods involved?	0.2		
	**Score methodological quality criteria**	**60%**		
2. Santini et al. (2023) [47]	S1. Are there clear research questions?	1		
	S2. Do the collected data allow to address the research questions?	1		
	5.1. Is rationale adequate for using a mixed methods design to address research question?	0.2		
	5.2. Are study components effectively integrated to answer the research question?	0.2		
	5.3. Are outputs of integration of qualitative & quantitative components adequately interpreted?	0.2		
	5.4. Are divergences & inconsistencies between quantitative & qualitative results adequately addressed?		X	
	5.5 Do the different study components adhere to the quality criteria of each tradition of the methods involved?			X
	**Score methodological quality criteria**	**60%**		
**Mean score of all studies**	S1. Are there clear research questions?	1		
	S2. Do the collected data allow to address the research questions?	1		
	5.1. Is rationale adequate for using a mixed methods design to address research question?	0.20		
	5.2. Are study components effectively integrated to answer the research question?	0.20		
	5.3. Are outputs of integration of qualitative & quantitative components adequately interpreted?	0.10		
	5.4. Are divergences & inconsistencies between quantitative & qualitative results adequately addressed?	0.00		
	5.5 Do the different study components adhere to the quality criteria of each tradition of the methods involved?	0.10		
	**Score methodological quality criteria**	**60%**		

* Supporting comments can be obtained from corresponding author.
