# Supplementary material for: The Impact of Digital Technology on the Physical Health of Older Workers: Scoping Review
Source: JMIR Aging. 2025 Nov 18;8:e78406. doi: 10.2196/78406 (PMC12673309; doi:10.2196/78406)
Supplement: Multimedia Appendix 4 [file aging_v8i1e78406_app4.pdf]

Multimedia Appendix 4. PRISMA-ScR checklist.

Table of contents

Study details according to the PRISMA-ScR checklist [49]. 2
Item 1. Title 2
Abstract 2
Item 2. Structured summary 2
Item 3. Rationale 2
Item 4. Objectives 2
Methods 3
Item 5. Protocol and registration 3
Item 6. Eligibility criteria 3
Item 7. Information sources 3
Item 8. Search 3
Item 9. Selection of sources of evidence 3
Item 10. Data charting process 4
Item 11. Data items 4
Item 12. Critical appraisal of individual sources of evidence 5
Item 13. Summary measures 5
Item 14. Synthesis of results 5
Item 15. Risk of bias across studies 5
Item 16. Additional analyses 5
Results 5
Item 17. Selection of sources of evidence 5
Item 18 Characteristics of sources of evidence 5
Item 19 Critical appraisal within sources of evidence 5
Item 20 Results of individual sources of evidence 5
Item 21 Synthesis of results 5
Item 22 Risk of bias across studies 5
Item 23 Additional analyses 5
Discussion 6
Item 24 Summary of evidence 6
Item 27: Funding 6
References 6

Study details according to the PRISMA-ScR checklist [49].

Item 1. Title

The impact of digitalization on the physical health of older workers: A scoping review.

Abstract

Item 2. Structured summary

Background: Digital technologies are increasingly present in workplaces, yet their impact on the physical health of older workers remains unclear.

Objective: This scoping review aims to examine and summarize the scientific evidence on how digital technology affects the physical health of older workers.

Methods: Following PRISMA-ScR guidelines, we conducted a scoping review of English-language peer-reviewed articles extracted from MEDLINE, Cochrane, Proquest, Web of Science, Scopus, APA PsycInfo, and ERIH PLUS. The review followed the Population, Concept, and Context (PCC) framework, including studies on workers aged 50 years or older, any form of digital technology (e.g. teleworking and the use of digital tools at work), and its impact on physical health (e.g. vision loss, musculoskeletal disorders). Studies that focused only on mental health were excluded. A 13-member research team screened studies in three stages: title and abstract screening, full-text review, and data extraction. Each article was independently reviewed by at least two researchers, within disagreements resolved through discussion. Data extraction and synthesis were conducted using the web-based systematic review platform Covidence.

Results: 18 studies were selected, published between 2012 and 2024, with most conducted in Europe (8) and Asia (6), followed by North America (2), Oceania (1), and Africa (1). We identified six key physical health areas impacted by digital technology in older workers: eye health, musculoskeletal health, metabolic and cardiovascular health, workplace sound levels, and user experiences of new technologies. Findings showed mixed effects, with notable negative impacts on eye strain, musculoskeletal disorders, and hearing health issues, but positive effects on weight management, cardiovascular health, physical activity, and perceived physical well-being.

Conclusions: Digital technology presents both risks and benefits for the physical health of older workers. While prolonged screen use and digital work environments contribute to eye strain, musculoskeletal issues, and hearing concerns, other technologies support better weight management, cardiovascular health, and increased physical activity. These findings also underscore the need for workplace intervention to reduce health risks.

Introduction

Item 3. Rationale

The aim of this scoping review is to locate and synthesize evidence on the impact of digital technology on the physical health of older workers.

Item 4. Objectives

This scoping review aims to examine and summarize the scientific evidence on how digital technology affects the physical health of older workers, focusing on the following topics:

1) Study design and focus.

2) Digital technology type

3) Employment setting

4) Physical health effects

5) Evidence gaps in this field.

Methods

Item 5. Protocol and registration

Previously, protocol of the scoping review was published [27] and registered under the International Registered Report Identifier (IRRID): PRR1-10.2196/59900 [28] before conducting the full electronic literature search.

Item 6. Eligibility criteria

The eligibility criteria for this scoping review are based on the Population, Concept and Context (PCC) criteria (see also Textbox 1 in main text):

Inclusion criteria
1. Population: older workers (50+ included in study)
2. Concept: digital technologies related to work
3. Context: physical health outcomes
4. Setting: nonclinical and in the work sphere
5. Study type: original studies with any design or data type (quantitative and qualitative)
6. Publication status: published in a peer-reviewed journal
7. Publication language: English
8. Full-text available

Exclusion criteria:

1. Population: younger workers (50+ not included in study)
2. Concept: digital technologies not related to work (e.g. for health management)
3. Context: non-physical health outcomes (e.g. mental health)
4. Setting: clinical and not in the work sphere
5. Study type: other study types (e.g. protocols, narrative reviews or systematic reviews)
6. Publication status: published without peer-review, dissertations, books, conference papers, letters, editorials.
7. Publication language: written in a language other than English
8. Full-text not available

Item 7. Information sources

The information sources for this scoping review include the following international bibliographic databases: MEDLINE, Cochrane, Proquest, Web of Science, Scopus, APA PsycInfo, and ERIH PLUS. For the protocol published in 2024 [27], the databases PubMed, PROSPERO, and JBI Evidence Synthesis were searched on April 15, 2024; however, no current or ongoing systematic or scoping reviews related to the topic of interest were identified. A preliminary search was also conducted around the same time using the databases MEDLINE, Cochrane, and Epistemonikos.

Item 8. Search

The syntax for the electronic search was developed and calibrated throughout March-April 2024 with the help from an experienced Research Librarian. The full search strategy is reported in the scoping review.

Item 9. Selection of sources of evidence

After importing references and removing duplicates on COVIDENCE, a web-based systematic review platform, a 13-member research team screened studies in three stages: title and abstract screening, full-text review to identify articles relevant to the research questions of our scoping review, and data extraction. Each article was independently reviewed by at least two researchers, within disagreements resolved through discussion. A pre-developed data extraction template was used to collect data from the selected articles, covering participant demographics, examined digital technologies, physical health outcomes and key findings. Regular online team meetings were held to ensure smooth progression through the different phases of article selection and the final data extraction phase.

Item 10. Data charting process

To organize and synthesize data effectively to extract information pertinent to our research question the data extraction was conducted entirely within COVIDENCE as it offers robust features for collaboration and comprehensive data handling. The data-charting form for data coding used for this was developed by JJAS and CMT and calibrated within the team. For each extracted article the data was coded independently by 2 researchers and final consensus was reached during online team meetings.

Item 11. Data items

Information from the following list of data items and their description will be retrieved from the articles to address the objectives of this scoping review. The name of the reviewer will be written at the top (for internal use only):

**Item**	**Description**
Author (APA style)	If 1 author: Author Surname (year). If 2 authors: Author and Author (year). If 3+ authors: First author et al. (year)
Year of data collection	
N (number of participants)	
N of participants invited	How many people were interviewed or had questionnaires sent to them in total, no matter if they responded?
N in follow-ups	For example, in intervention or randomized control studies: How many participants responded in a follow-up (e.g., after 1 year)?
Country	
Type of study	Quantitative, qualitative, mixed
Sampling method	How were participants recruited for the research?
Data collection method	What type of method was used? e.g. semi-structured interview, in-depth interview, face-to-face questionnaire/survey, telephone survey, online survey, mail survey
Data recording method	Pencil-paper, online, audio, video, , secondary data, other
Type of population	e.g. employees, employers, general population
Type of workers	Occupation/Employment branch
Age categories used in analysis/results	If no age categories were analyzed, the age range of the sample
Study includes both young and older workers?	Studies with young people may only be included if older workers are also represented and analysed (at least 50 years old)
Includes both older adults and older workers?	Studies with older adults who do not work may only be included if older workers are also analysed
Gender/sex used in analysis/results	If participants are described for each gender, check each box ("female", "male"). If participants are described as whole check "total f+m"
Digital tool	Name / describe the digital tool that was used
Digital tools characteristics	Implicit (e.g., remote work, hybrid), Explicit (e.g., apps, digital tools used at work), both (implicit and explicit)
Type of physical health	What physical health outcome was studied?
Instruments	Describe instruments/measures/assessments used for the variables digital tools and physical health. How were the variables assessed?
Main findings	Describe relevant findings for our research goals/questions
General effects	Positive, negative, mixed, no effects

Item 12. Critical appraisal of individual sources of evidence

Although critical appraisal of individual studies is not typically conducted in a scoping review [49], we assessed the quality of the 18 included studies using the Mixed Method Appraisal Tool (MMAT) [50] (see Multimedia Appendix 3). Any discrepancies were resolved through consensus between the reviewers.

Item 13. Summary measures

Not applicable for scoping reviews.

Item 14. Synthesis of results

The charted data is placed in a table (Multimedia Appendix 2) and narratively synthesized in the main text.

Item 15. Risk of bias across studies

Not applicable for scoping reviews.

Item 16. Additional analyses

Not applicable for scoping reviews.

Results

Item 17. Selection of sources of evidence

The number of sources of evidence screened, assessed for eligibility, and included in the review, with reasons for exclusions at each stage, can be found in the PRISMA flow chart (Figure 1, main text).

Item 18 Characteristics of sources of evidence

Eligibility was determined based on the PCC framework and additional criteria for inclusion and exclusion is provided in textbox 1 in the main text. A description of the characteristics of the included studies (period of data collection, year of publication, study design, geographic location, sample size of study, type of working population, digital technology and physical health outcome) is provided in the main text and summarised in Multimedia Appendix 2.

Item 19 Critical appraisal within sources of evidence

Study quality was assessed using the MMAT [50], which evaluates five study design categories for the sources of evidence: (1) qualitative studies, (2) quantitative randomized controlled trials, (3) quantitative non-randomized studies, (4) quantitative descriptive studies, and (5) mixed methods studies. The MMAT includes a two-part checklist: an initial screening with two questions to confirm the study's empirical nature (clarity of research questions and feasibility of addressing them), followed by five design-specific criteria guiding the assessment process. Results are provided in Multimedia Appendix 3 and further described in the main text.

Item 20 Results of individual sources of evidence

For each included source of evidence, the relevant data that relate to the review questions and objectives are charted in Multimedia Appendix 2.

Item 21 Synthesis of results

In the subsection "Physical health outcomes" located in the results section of the main text, the specific physical health outcomes associated with digital technology analysed in the selected studies are described.

Item 22 Risk of bias across studies

Not applicable for scoping reviews.

Item 23 Additional analyses

Not applicable for scoping reviews.

Discussion

Item 24 Summary of evidence

Addressing a research gap: While previous scoping and systematic reviews have predominantly focused on how digital technologies in the workplace affect mental health outcomes, such as technostress and burnout, this scoping review aimed to identify digital technologies that influence the physical health of older workers and identify occupational sectors most associated with these technologies.

Opportunities and risks of digital technologies: While integration of digital technologies into the workplace offers potential benefits, such as optimizing work processes, comprehensive studies exploring the direct causal links between digital work environments and physical health outcomes among older workers are scarce.

Thematic insights into physical health domains: The identified studies show how increasing integration of digital technologies into work environments might create both opportunities and challenges for the physical health of older workers related to eye health, musculoskeletal health, metabolic and cardiovascular health, workability and workplace sound levels.

Item 25 Limitations

A potential limitation of this scoping review is that, despite an extensive and systematic search across seven major academic databases, some relevant studies may have been missed (non-English language publications, very recent publications).

Item 26 Conclusions

The findings highlight a diverse range of physical health implications, both positive and negative, spanning eye health, musculoskeletal conditions, metabolic and cardiovascular health, and workplace ergonomics. The results underscore the pressing need for targeted workplace interventions and employer-driven initiatives to ensure a healthier work environment for ageing employees

Item 27: Funding

The research was partially financed through the COST Action CA21107 “Work inequalities in later life redefined by digitalization” (DIGI-net) that is supported by the European Cooperation in Science and Technology (COST) (https://www.cost.eu/actions/CA21107/).

References (as listed in full paper)

27. Spijker JJA, Barlın H, Grad DA, Gu Y, Klavina A, Korkmaz Yaylagul N, et al. The Impact of Digital Technology on the Physical Health of Older Workers: Protocol for a Scoping Review. JMIR Research Protocols. 2024;13:e59900. https://doi.org/10.2196/59900.
28. Spijker JJA, Barlın H, Grad DA, Gu Y, Korkmaz-Yaylagul N, Kulla G, et al. The Impact of Digitalization on the Physical Health of Older Workers: Scoping Review Protocol Summary. OSF Registries [Internet]. 2024. Available from: https://osf.io/dj34a.
49. Tricco AC, Lillie E, Zarin W, O'Brien KK, Colquhoun H, Levac D, et al. PRISMA Extension for Scoping Reviews (PRISMA-ScR): Checklist and Explanation. Annals of Internal Medicine. 2018;169(7):467-73. https://doi.org/10.7326/M18-0850.
50. Hong QN, Fàbregues S, Bartlett G, Boardman F, Cargo M, Dagenais P, et al. The Mixed Methods Appraisal Tool (MMAT) version 2018 for information professionals and researchers. Education for information. 2018;34(4):285-91. https://doi.org/10.3233/EFI-180221.
